# Social Instability in Laying Quail: Consequences on Yolk Steroids and Offspring's Phenotype

**DOI:** 10.1371/journal.pone.0014069

**Published:** 2010-11-22

**Authors:** Floriane Guibert, Marie-Annick Richard-Yris, Sophie Lumineau, Kurt Kotrschal, Daniel Guémené, Aline Bertin, Erich Möstl, Cécilia Houdelier

**Affiliations:** 1 UMR 6552 Ethologie animale et humaine, CNRS-Université de Rennes 1, Rennes, France; 2 Konrad-Lorenz-Forschungsstelle, University of Vienna, Grünau, Austria; 3 UR83 Unité de Recherches Avicoles, INRA, Nouzilly, France; 4 Syndicat des Sélectionneurs Avicoles et Aquacoles Français, INRA Tours-Nouzilly, UR83-URA, Nouzilly, France; 5 UMR 85 Physiologie de la reproduction et des comportements, INRA-CNRS-Université de Tours-Haras Nationaux, Nouzilly, France; 6 Department of Natural Sciences - Biochemistry, University of Veterinary Medicine, Vienna, Austria; Pennsylvania State University, United States of America

## Abstract

Individual phenotypic characteristics of many species are influenced by non-genetic maternal effects. Female birds can influence the development of their offspring before birth via the yolk steroid content of their eggs. We investigated this prenatal maternal effect by analysing the influence of laying females' social environment on their eggs' hormonal content and on their offspring's development. Social instability was applied to groups of laying Japanese quail females. We evaluated the impact of this procedure on laying females, on yolk steroid levels and on the general development of chicks. Agonistic interactions were more frequent between females kept in an unstable social environment (unstable females) than between females kept in a stable social environment (stable females). Testosterone concentrations were higher in unstable females' eggs than in those of stable females. Unstable females' chicks hatched later and developed more slowly during their first weeks of life than those of stable females. The emotional reactivity of unstable females' chicks was higher than that of stable females' chicks. In conclusion, our study showed that social instability applied to laying females affected, in a non-genetic way, their offspring's development, thus stressing the fact that females' living conditions during laying can have transgenerational effects.

## Introduction

Variations among individual phenotypes come from the genetic background of individuals, but also from influences of various non-genetic factors occurring during their development. Mothers' influences are one of the non-genetic factors that play a fundamental role in this variability. During the mothering phase, mothers influence not only the neurobiological development of their young (through DNA methylation patterns, chromatin marking systems, hormones) [1], but also their behavioural development by influencing their emotive and social traits [2]–[6], their sexual and maternal behaviours [7]–[9], their cognitive abilities [10] and their biological endogenous rhythms [11]. Non-genetic maternal influences can also occur during the prenatal period.

In mammals, the social status and the social environment of pregnant females strongly affect their offspring's morphological and behavioural development [12]–[14]. For example, in wild spotted hyenas (*Crocuta crocuta*), the cubs of dominant females show higher rates of aggression and mounting behaviour than the cubs of subordinate females [15]. These behavioural differences may be linked to differences in plasmatic testosterone levels between dominant and subordinate females, testosterone levels during late gestation being higher in dominant than in subordinate females [15]. Moreover, changes in females' social environment (due to social instability, crowding, agonistic social encounters) during pregnancy can increase their offspring's emotional reactivity, impair their learning abilities and alter their social, sexual and maternal behaviours [12]–[14]. These effects on offspring are thought to be mediated by modifications of glucocorticoid and androgen plasmatic concentrations in pregnant females [13], [16], [17].

Reports also show that avian mothers can prenatally affect their offspring's phenotypes through the modulation of egg hormonal levels [18]–[20]. Among egg hormonal components, testosterone has been found to vary according to the birds' social context and to affect chicks' growth and behaviour. Breeding density [21]–[23], [19], frequency of social intrusions [24] or of social aggression [25], maternal social status [26], [27] and male attractiveness [28]–[30] are social factors that can affect yolk androgen levels. Moreover, artificial increase of yolk testosterone levels influence hatching time [31]–[33], post-hatching growth [31], [34], immune functions [35], [36], early survival [31], [32] and behaviour [19], [20], [37], [38] of chicks. However, effects of artificial increase of yolk testosterone levels on chick's development appear to vary among species and studies. Thus, injection of testosterone into the yolk can bring forward the hatching time of black-headed gulls' (*Larus ridibundus*) chicks [32], [33] or delay it in American kestrels (*Falco sparverius*) [31]. High levels of yolk testosterone can stimulate post-hatching growth in starlings (*Sturnus vulgaris*) [34] or reduce it in American kestrels [31]. Finally, yolk testosterone injections can make Japanese quail's (*Coturnix coturnix japonica*) chicks either more [38] or less fearful [37]. These discrepancies may be due to species-specific characteristics or to the hormonal administration method, as injection into the yolk does not necessarily mimic natural maternal variations [39]. Although hormonal injections in eggs enable us to better understand how yolk testosterone can affect chicks' development [40], variability in the results obtained to date indicates that data on what happens under more “natural” situations are needed. In other words, we need to know how egg hormonal levels and how chicks' characteristics are affected by the environment of laying females. A more “naturalistic” approach should therefore help us understand the adaptive significance of prenatal maternal effects.

In this context, our study aimed at analysing the effects of the social environment of laying females on (1) yolk steroid levels in their eggs and (2) the general development of their chicks. To date, studies of prenatal maternal effects in birds have mainly focused on altricial and semi-precocial species, leaving a gap in knowledge about what happens in precocial species. We thus studied a precocial species, the Japanese quail. We compared the eggs and the offspring of females living either in a stable social group or in an unstable social group (with regularly changing members). Previous studies showed that quail can maintain close relationships with conspecifics and are able to discriminate familiar conspecifics from unknown conspecifics [41], [42]. Moreover, like many other bird species, they show physiological and behavioural responses to modifications of their social environment [43]–[48]. We therefore hypothesized that social instability in laying females' groups would influence their yolk steroid levels and would modify the somatic and behavioural development of their offspring.

## Materials and Methods

### Ethics Statement

All experiments were approved by the departmental direction of veterinary services (Ille et Vilaine, France, Permit number 005283) and were performed in accordance with the European Communities Council Directive of 24 November 1986 (86/609/EEC).

### Laying females

**Figure 1:**
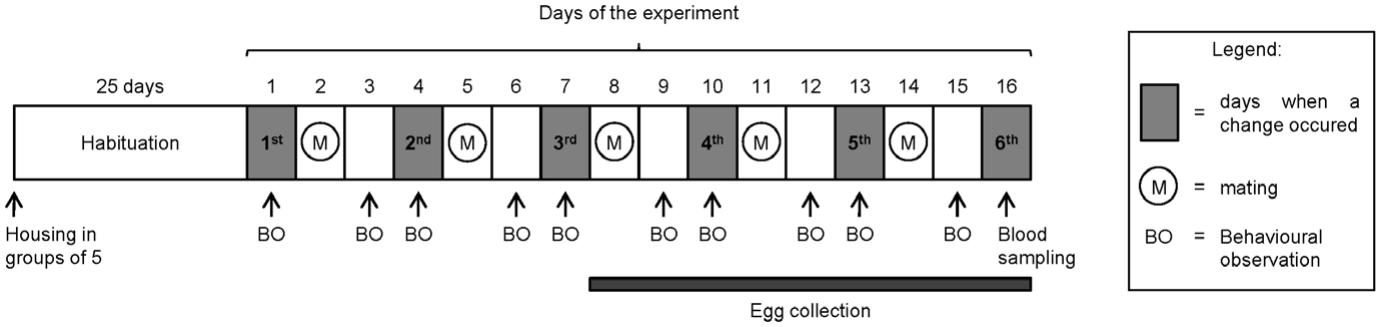
Timing of the experiment on females.

The timing of the experiment is summarised in figure 1.

#### Housing

One hundred female Japanese quail from a commercial line were split into 20 groups of 5 individuals when 6 weeks old. Each group was housed in a separate cage (100 cm×70 cm×62 cm), but in the same room. Birds had access to food and water *ad libitum*. They were exposed to a 14∶10 h light∶dark cycle and to a temperature of 19±1°C.

#### Social instability procedure

After 25 days of habituation to their housing conditions, the female groups were randomly assigned to two experimental sets: 10 groups were assigned to the stable (S) set and the other 10 to the unstable (UNS) set. For the UNS set, the group composition was changed every 3 days over a 16-day period (6 changes in all). A computer program picked two females in each group to be moved to another unstable group. These two females were called the UNS unfamiliar females, and the three females who stayed in their home cage were called the UNS resident females. Selection of females by the computer program ensured the arrival of unfamiliar birds after each change and meant that each UNS female was alternately unfamiliar and resident during the experiment. The S set females stayed in the same group throughout the experiment. To control the disturbance due to human manipulation during these changes, females of the two experimental sets were taken out of their home cage during the change. The manipulation consisted in taking all the birds out of their cage and placing them in a box (48×33×15 cm) equipped with net openings and wood shavings on the floor. Boxes were familiar to the females as females had been habituated daily to short stays in these boxes before the beginning of the social instability procedure. After 5 minutes in the box, females were moved back, either in their home cage (S females and UNS resident females), or in another cage (UNS unfamiliar females). The UNS unfamiliar females were gently marked with a small paint spot on their back to distinguish them from the resident females. Changes occurred in the morning, 1 hour after the lights were switched on. By the end of the experiment, all unstable females had been moved at least twice.

#### Mating

The day following a change, females were presented individually with a male for mating (5 mating sessions in all). A group of mature males (N = 20) was used for the two experimental female sets. Two males were always associated with the same two groups of females (an S and an UNS group) and met them alternately. Pairs stayed together in a small cage until copulation had occurred.

#### Behavioural and physiological measures

Through direct observations of the groups just after each change and 48 hours later (the day before the next change), the impact of the procedure on the females' behaviour was evaluated. Every 6 minutes, for 1 hour, the observer instantaneously recorded agonistic behaviours (attacks, pecks, avoidances) within each group (i.e. 10 observations per group) using the scan-sampling method [49], [50].

Corticosterone levels were assayed to evaluate the impact of social partners' change on the females' physiology. Blood samples were collected by decapitation 30 minutes after the last change and within the first minute after capture (in order to avoid the effects of capture on hormone levels [51]). Decapitation appears to affect basal corticosterone levels much less than venipuncture, cardiac or jugular puncture [52]. Blood samples were collected in tubes containing EDTA (2 mg/ml blood), just after the last change, from 10 S set females (one female per group) and 20 UNS set females (two females per group, one unfamiliar and one resident). The blood samples were then centrifuged at 2000 g for 15 min at 4°C. The plasma was collected and stored at −20°C prior to the measurement of corticosterone levels using a specific radioimmunoassay [53].

### Eggs

#### Egg collection and incubation

During the experiment, the laying rate stayed high in all groups with almost similar laying rates for the two sets (3.58±0.20 eggs/cage/day for the UNS set and 4.12±0.21 eggs/cage/day for the S set; Mann Whitney *U*-test, *U* = 25.5, p = 0.063).

As the formation of individual yolks lasts 7 days [54], fertilised eggs from each group were collected from the 7^th^ day after the first change. They were collected daily for 9 days and identified according to the group they came from (as we could not know from which female each egg came). During collection, 36 eggs from the S set and 39 eggs from the UNS set (3.6±0.4 eggs per S group and 3.9±0.4 eggs per UNS group) were stored at −20°C for later steroid hormone analyses. Most of the collected eggs (354 eggs from the S set and 314 eggs from the UNS set, i.e. 35.40±2.50 eggs per S group and 31.40±2.51 eggs per UNS group) were placed in an incubator for 18 days. During the first 15 days, eggs were maintained at 37.7°C with a relative humidity of 45% and an automatic rotation of 45° twice a day. For the last 3 days, the temperature was decreased to 37.2°C, the humidity was raised to 60% and egg rotation was stopped to favour hatching.

#### Yolk steroid analysis

Steroid extraction and assays (enzyme immunoassay) followed a method similar to that described by Möstl *et al.* [55] for chicken eggs and by Hackl *et al.* [56] and Bertin *et al.* [57] for quail eggs. For steroid extraction, the frozen yolk was separated from the eggshell and the albumin, as described by Lipar *et al*. [58] and Hackl *et al*. [56], and weighed. As the distribution of hormones varies between egg layers [55], [56], [58], the entire yolk was mixed before being assayed. Each yolk was suspended in 10 ml of water and vortexed twice for 30 s. Samples were then stored overnight at 4°C. Samples were then vortexed and 1 ml of the suspension was transferred into a new vial. The suspension was then diluted with 4 ml methanol, vortexed for 30 min and stored at−20°C overnight to precipitate apolar lipids. Samples were then centrifuged (−10°C, 2500 g, 10 min). 10 µl of the supernatant were transferred into a new vial, dried under a stream of nitrogen at 60°C and then dissolved in 500 µl EIA buffer. The extract was diluted 1∶5 with EIA buffer. 10 µl of this last solution were used for testosterone and androstenedione assays. For progesterone assay, we used 10 µl of the solution after an additional 1∶10 dilution. For full descriptions of antibodies and validation of enzyme immunoassays, see Palme and Möstl [59]; Hirschenhauser *et al*. [60]; Möstl *et al*. [55]. We measured yolk testosterone in 7 assays, androstenedione in 8 assays and progesterone in 7 assays. The inter-assay coefficients of variation were 14.2%, 8.7% and 10.1% respectively for the low-level pool and 5.2%, 8.7% and 7.1% for the high-level pool. The intra-assay variation was 8.5%, 4.2% and 9.2% respectively.

### Chicks

#### Housing

At hatching, chicks were identified according to the group they came from, stable (S) or unstable (UNS), and individualised using coloured leg rings. Chicks were then housed in groups of 8 from the same experimental set but from different mother groups. Thus, 10 S groups (N = 80 S chicks) and 10 UNS groups (N = 80 UNS chicks) were formed. We chose to house chicks from the same experimental set together because housing chicks of different sets in the same cage could influence the results. Indeed, growth and behavioural differences related to the different treatments may, via social interactions, exaggerate or induce other differences that are not directly due to the effects of the treatment [61].

A warming bulb (38±1°C) was placed in each cage to ensure chicks' thermoregulation until they were 10 days old. After this, when chicks were able to regulate their own temperature, the warming bulbs were switched off and the temperature in the room was kept at 20±1°C. Chicks were exposed to a 10∶14 h light∶dark cycle. Water and food were provided *ad libitum.* The general development of chicks was followed by weighing them weekly, from hatching until they were 4 weeks old, using electronic scales.

#### Behavioural tests

Classical ethological tests for poultry, based on different social and potentially fearful situations, were used to assess the general emotional reactivity of chicks [62]. Fifty chicks from the S set (5 per group) and 50 chicks from the UNS set (5 per group) were tested in the tests described below.

Emergence test: Chicks tested in the emergence test were 14 to 15 days old. This test followed a protocol similar to that described by Jones *et al.* [63]. The test chick was taken from its home cage to a dark room in a cardboard box (18×18×18 cm). The cardboard box containing the chick was placed on the left side of a wooden box (83×60×35 cm). The floor of the wooden box was covered with wood shavings and the side of the wooden box facing the experimenter was a glass window. The cardboard box was kept closed for 1 min. The cardboard box was then opened and the lights switched on. The experimenter recorded the latency of emergence into the new environment for each chick. A chick was given a maximum of 3 min to leave the cardboard box and, if it had not emerged after these 3 min, a maximum score of 180 s was recorded. Unwillingness to enter novel, exposed areas is common throughout the animal kingdom, and time taken to emerge from a sheltered area into an open and unfamiliar environment has been measured in laboratory rodents, domestic chicks and quail under the assumption that fearful or timid animals take longer to emerge [64]–[66].

Separation test: When they were 10 days old, chicks were tested in a separation test in their familiar environment. Our protocol was adapted from that described by Launay [67] and Bertin & Richard-Yris [2]. In this test, the test chick was isolated in its home cage by removing its 7 cage mates. Latency of first call, latency of first step, number of calls and number of steps made by the chick were then recorded during 3 min. This test, which does not involve any aspect of environmental novelty, evaluated the reactivity of chicks to social separation. The number of calls and the number of steps are considered to be positively correlated to the motivation to join conspecifics (i.e. social motivation, see [67]).

Open-field test: Chicks were tested in the open-field when they were 17 to 18 days old. The test chick was taken from its home cage to a dark room in a cardboard box. The chick was then put in the centre of a wire-netting cylinder (Ø120 cm, H 70 cm) with a linoleum floor. Lights were then switched on and the experimenter, hidden behind a two-way mirror, recorded latency of first call, latency of first locomotor act (first walk or run), number of calls, locomotor acts, exploratory acts (floor and wire-netting pecking) and high-posture observations made by the chick during 5 min. The open-field test is a well-validated test in birds that evaluates both the general/antipredatory fear of the animal and its response to social isolation/dependence [62]. Behavioural responses in this test are linked to these two motivations. Longer latencies to move and lower preening and pecking frequencies are indications of high general fear, whereas more jumps, more vocalisations and shorter latencies to emit calls are more related to social isolation [62]. General locomotion is generally interpreted within the framework of fear of novelty. Indeed, less locomotion, especially with freezing, reflects a high level of fearfulness, the animal then using a passive strategy to challenging the situation [62]. However, reports show that high levels of locomotion can also reflect high fear levels in the arena, individuals using in this case an active strategy of response (escape behaviour and/or active search for conspecifics) [68]–[71].

### Statistical analyses

Kolmogorov-Smirnov tests were used to determine whether data sets were normally distributed. Females' data (frequencies of agonistic behaviour and corticosterone plasmatic levels) and chick's hatching dates and behavioural data were not normally distributed. Mann-Whitney *U*-tests were therefore used. Means per group were used to analyse egg hormonal contents. Yolk-hormone data were log-transformed (Y+1) and analysed with a MANOVA and individual one-way ANOVAs. Chick-weight data were analysed using a one-way repeated measures ANOVA (procedure x age) and subsequent post-hoc Fisher LSD tests. Data are represented as means ± standard error of the mean (SEM). All analyses were performed using Statview Software (SAS, Cary, NC), with significance set at *p*≤0.05.

## Results

### Effects of social instability on females

Our instability procedure modified social interactions between females. After a change, UNS females were more aggressive towards one another than were S females (respectively: 1.50±0.37 and 0.22±0.13 agonistic behaviours per observation; Mann-Whitney *U*-test, *U* = 9.5, *p* = 0.0017). This difference tended to persist 48 h later, although it was no longer significant (0.88±0.23 agonistic behaviours per observation for UNS females and 0.38±0.17 for S females; *U* = 26, *p* = 0.062).

**Figure 2:**
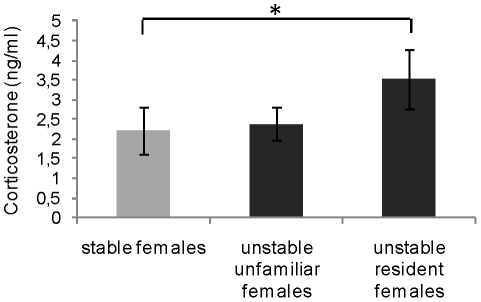
Corticosterone levels in stable and unstable females. Mean ± SEM plasmatic corticosterone concentrations (ng/ml) in stable females (N = 10) (grey bar) and unstable unfamiliar (N = 10) and resident females (N = 10) (black bars) after the last change. Mann-Whitney *U*-test, *p<0.05.

After the last change, plasmatic corticosterone levels tended to be higher in UNS females than in S females (respectively, 2.95±0.43 ng/ml (N = 20) and 2.20±0.59 ng/ml (N = 10); *U* = 61, *p* = 0.086). Plasmatic corticosterone levels of UNS resident females were significantly higher than those of S females (*U* = 23, *p* = 0.041; figure 2), whereas levels did not differ significantly between UNS unfamiliar females and S females (*U* = 38, *p* = 0.36; figure 2). Differences between UNS resident females and UNS unfamiliar females were not significant (*U* = 31, *p* = 0.15; figure 2).

### Effect of social instability on yolk hormone levels

**Figure 3:**
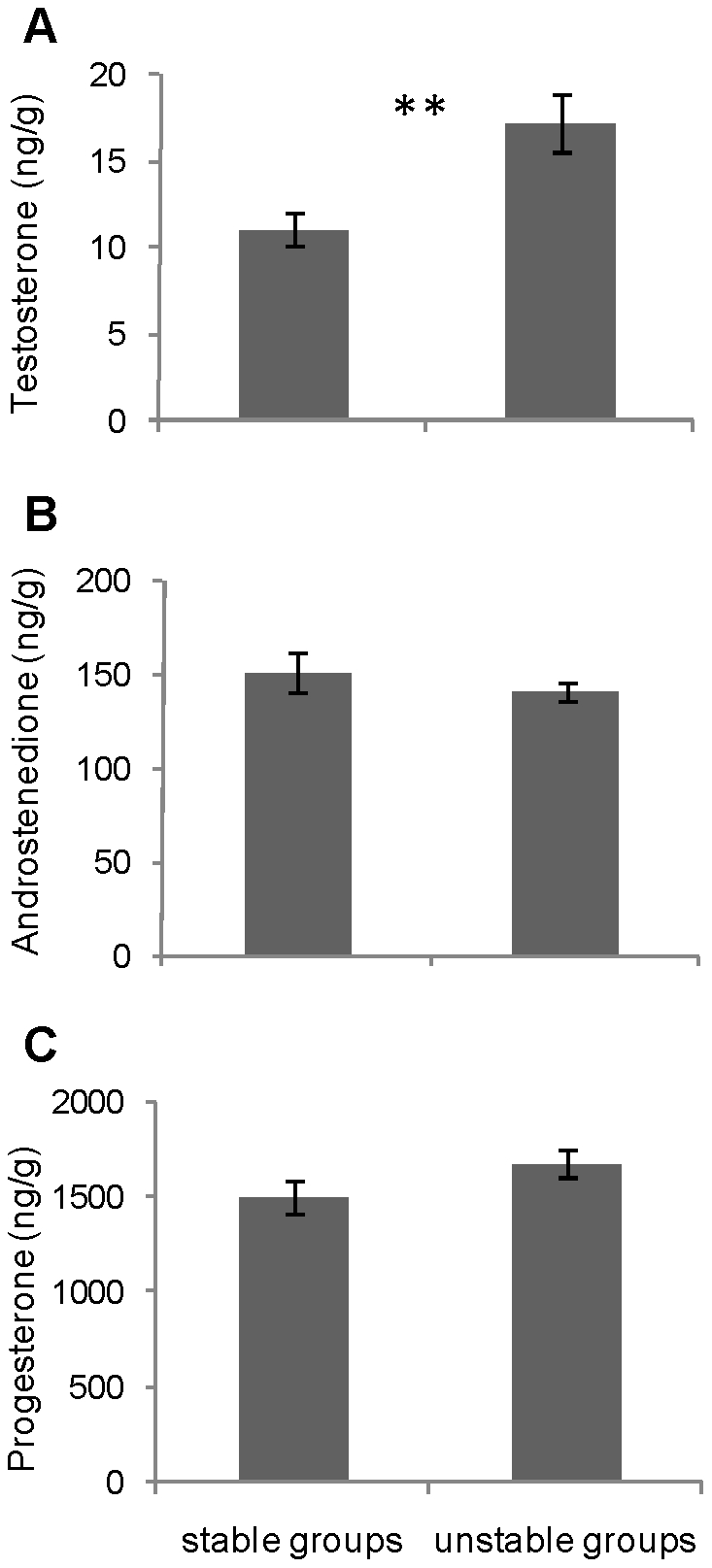
Yolk steroid levels in stable and unstable females' eggs. Mean ± SEM yolk testosterone (**A**), androstenedione (**B**) and progesterone (**C**) concentrations (ng/g) in eggs of stable and unstable females. One-way ANOVA, **p<0.01.

A significant overall effect on yolk hormone concentrations was found (Manova, *F*_3,16_ = 6.442, *p* = 0.0046). The yolks of UNS females' eggs contained higher testosterone concentrations than did the yolks of S females' eggs (*F*_1,18_ = 11.042, *p* = 0.0038; figure 3A). No significant differences could be evidenced for androstenedione (*F*_1,18_ = 0.537, *p* =  0.47; figure 3B) and for progesterone (*F*_1,18_ = 2.464, *p* = 0.13; figure 3C).

### Effect of prenatal social instability on chicks' phenotypes

#### Effects on hatching and growth

UNS chicks hatched later than did S chicks (respectively, 18.44±0.06 and 18.29±0.05 days until hatching; Mann-Whitney *U*-test, *U* = 2660.5, *p* = 0.044).

We observed significant effects of our procedure (repeated measures ANOVA, *F*_1,158_ = 3.865, *p* = 0.05) and of age (*F*_4,632_ = 12246.9, *p*<0.0001) and a significant interaction between procedure and age (*F*_4,632_ = 3.102, *p* = 0.015) on chicks' growth. Although, at birth, the weights of the chicks in the two sets were similar (post-hoc Fisher LSD test, *p* = 0.92; table 1), growth of UNS chicks appeared to be delayed during their first 3 weeks of life compared to that of S chicks (*p* = 0.0038 for 1-week-old chicks; *p* = 0.0086 for 2-week-old chicks; *p* = 0.024 for 3-week-old chicks; table 1). However, the weights of 4-week-old chicks of the two sets were similar (*p* = 0.51; table 1).

**Table 1:** Body weight of stable and unstable chicks from hatching to 4 weeks old.

	Body weight (g)	
Age	Stable chicks	Unstable chicks
hatching	9.80±0.10	9.79±0.12
1 week	42.20±0.71	39.27±0.70 **
2 weeks	94.25±1.29	89.10±1.44 **
3 weeks	151.83±1.83	145.56±2.05 *
4 weeks	202.56±2.07	200.50±2.31

Mean ± SEM; Post-hoc Fisher LSD test,

* *p*<0.05;

** *p*<0.01.

#### Effects on behavioural characteristics

In emergence tests, UNS chicks left the box later than did S chicks (respectively, 22.92±6.04 s and 11.12±2.69 s; *U* = 939.5, *p* = 0.031).

In separation tests, UNS chicks made their first step quicker than did S chicks (respectively, 6.72±1.43 s and 25.66±9.02 s; *U* = 957.5, *p* = 0.039). However, numbers of steps did not differ significantly between the two sets (S chicks: 117.80±11.60 steps; UNS chicks: 134.22±11.85 steps; *U* = 1097.5, *p* = 0.29). Neither latencies of first call (S chicks: 22.20±10.08 s; UNS chicks: 14.36±6.22 s; *U* = 1177.5, *p* = 0.61) nor numbers of calls (S chicks: 82.02±6.85; UNS chicks: 85.34±6.48; *U* = 1160.5, *p* = 0.54) differed significantly between the two sets.

**Figure 4:**
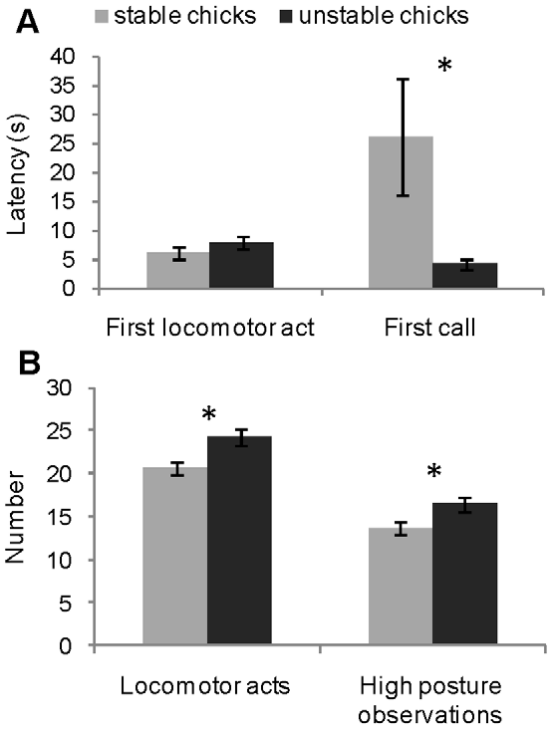
Behaviour of stable and unstable chicks in open-field tests. **A.** Mean ± SEM latencies of first locomotor act and of first call during open-field tests for stable and unstable chicks. **B.** Mean ± SEM numbers of locomotor acts and of high-posture observations during open-field tests for stable and unstable chicks. Mann-Whitney *U*-test, *p<0.05.

Although UNS chicks did not differ from S chicks regarding latency of first locomotor act in open-field tests (*U* = 1019.5, *p* = 0.11; figure 4A), they made more locomotor acts during the test (*U* = 901.5, *p* = 0.016; figure 4B). They emitted their first call sooner than did S chicks (*U* = 950, *p* = 0.035; figure 4A), but they did not emit more calls (respectively, 173.86±8.46 and 153.32±9.60 calls; *U* = 1061.5, *p* = 0.19). UNS chicks made more high-posture observations than did S chicks (*U* = 894, *p* = 0.014; figure 4B). Numbers of exploratory acts did not differ significantly between UNS chicks and S chicks (respectively, 4.52±0.71 and 5.54±0.77; *U* = 1095, *p* = 0.28).

## Discussion

Here, we demonstrated that social instability in groups of female quail influenced their behaviour, the hormonal content of their eggs and the somatic and behavioural development of their offspring.

During this experiment, females from unstable groups expressed more agonistic interactions than did females from stable groups. A similar enhanced aggression level was previously found in groups of male Japanese quail subject to social instability [43], [45]. The persistence of agonistic behaviour 48 h after a change suggests that the effects of our social disturbance remained important throughout the experiment. Contrary to behavioural data, the analysis of corticosterone plasmatic levels showed no clear effect of these experimental conditions on the females' physiology. Differences in corticosterone concentrations were found only between unstable resident females and stable females. This indicates that hormonal responses during encounters may vary according to the individual status and may reflect different styles of coping with social stress [72]. More frequent blood samplings would therefore be necessary to show a clear effect of the treatment on plasma corticosterone levels in females. However, in our study, females of the unstable set were alternately resident and unfamiliar during the experiment, and were therefore subject to the same global treatment.

Behavioural and physiological responses of quail to changes in their social environment have previously been demonstrated [43]–[48]. Young as well as adult quail are highly sensitive to social separation and they are able to discriminate a familiar conspecific from an unfamiliar conspecific, preferring to stay close to familiar quail [41], [42], [71], [73]. Moreover, adult birds are less aggressive in stable groups than in unstable groups (our study for females; [43], [45] for males). Despite a rather good knowledge of quail's social behaviour, the social habits of quail in the wild are still not well known. This is mainly due to the fact that this migratory bird is small, cryptic and hard to observe in its natural environment (wide open landscapes and large cultivated fields) [74]. Observations of feral populations of Japanese quail or populations housed in a semi-natural environment, and studies on wild population of a sibling species, the European quail (*Coturnix coturnix*), indicate that quail aggregate in large, loose colonies during the wintering phase and can maintain close relationships with conspecifics [74]. In reproductive areas, quail show an aggregative pattern of spatial distribution, without territorial boundaries, on restricted traditional sites [75], [76]. Quail appear to be a successive monogamous species (pairs stay close together only during the pre-laying and laying phases) [77]. However, even though pairs tend to remain at a distance from other pairs, they stay spatially close and all pairs follow more or less the same daily routes [78]. As quail are able to discriminate familiar from unknown conspecifics [42], [73], we assume that, in a reproductive area, all quail know one another and may thus form a “stable group”. During the reproductive phase, different quail groups may arrive in succession on traditional reproduction sites, attracted by the presence of others [76]. Thus, on these sites, the arrival of new breeders could induce social perturbation for the quail that were already present.

Our social instability procedure had no effect on egg androstenedione and progesterone contents but affected testosterone levels. Unstable females laid eggs with more testosterone than did stable females. Reports reveal a similar impact of social living conditions on egg testosterone levels in other species. Thus, eggs of house sparrows (*Passer domesticus*), American coots (*Fulica Americana*) and European starlings breeding in a high-density colony have been shown to contain more testosterone than did those of females nesting in isolation or near only a few conspecifics [21], [22], [79]. Authors of these studies suggested that, in high-density colonies, birds express more aggressive interactions in relation to territory defence. Similarly, testosterone levels in free-living tree swallows' eggs appear to be positively correlated to the levels of females' aggressive interactions [25]. As increased aggression levels can lead to elevated circulating levels of androgens in females [80], increase of yolk testosterone levels could be directly correlated to the increase of this hormone in the plasma of females. Indeed, in some studies, plasma and yolk steroids levels appear to be positively correlated [81], [82]. However, other studies reported either negative [24], [83] or no correlation [84]–[86] between testosterone levels in plasma and eggs. Groothuis and Schwabl [40] proposed three alternative mechanisms that could explain steroid accumulation in the eggs. The *physiological epiphenomenon hypothesis* implies that concentrations of maternal steroids in the yolk are a consequence and reflection of general maternal hormone production, and predicts a positive correlation between steroid concentrations in a female and her eggs. The *flexible distribution hypothesis* implies a regulation of the distribution of produced hormones between maternal circulation and yolk and would explain a negative correlation between ovarian hormones in eggs and females' plasma. Finally, the *independent regulation hypothesis* implies that hormone concentrations in eggs and maternal circulation are regulated independently, and predicts the absence of correlation between hormonal concentrations in females and eggs. Current data do not allow us to favour one of these hypotheses. Further research will thus be necessary to understand the mechanisms involved in the modulation of yolk maternal steroids.

Our social instability procedure applied to laying females affected the general development of their offspring. Embryonic growth and postnatal weight-gain of unstable females' chicks were impaired. Moreover, unstable chicks presented greater emotional reactivity than did stable chicks. Indeed, in emergence tests, they took longer to leave the emergence box, indicating a more cautious behaviour when facing a novel environment [64]–[66]. In open-field tests, unstable chicks again appeared more disturbed and expressed more locomotor activity. This important locomotor activity could be interpreted as an attempt to escape [69], [70] or as a search for conspecifics following social separation [68], [71]. In both cases, this reveals that the situation is highly disturbing. In addition, unstable chicks called sooner and presented more high-posture observations, reflecting a stronger reaction to social separation, with more behavioural attempts to search for conspecifics [62], [71]. This sensitivity to social separation was also observed in separation tests, as unstable chicks made their first step quicker than did stable chicks. In general, chicks of unstable females showed a higher inherent fearfulness than did those of stable females.

Differences between our two sets of chicks may be linked to differences in yolk testosterone levels. Our results agree with reports showing, in diverse species, that an increase of yolk testosterone by injecting eggs delays hatching and reduces growth rates [31], [61], [87], [88]. However, other studies report positive effects of testosterone injections in yolks on embryonic muscular development, hatching date and postnatal growth [32]–[34], [89], [90]. Similarly, yolk testosterone levels can induce contradictory results regarding the behavioural development of quail chicks. Thus, Daisley *et al*. [37] found that quail chicks from eggs with higher testosterone concentrations were less fearful and less socially dependant (“proactive phenotype”), whereas Okuliarová *et al*. [38] and Bertin *et al*. [57] found that chicks exposed to higher yolk testosterone concentrations were more fearful. These contradictory results could be explained by two factors. First, a dose-dependent effect of testosterone may be involved. Hormones' dose-response curves are often non-monotonic, and present an inverted U-shape, with intermediate doses having greater effects than either higher or lower doses [40]. For example, low doses of yolk testosterone have been shown to increase skeletal growth in eastern bluebirds (*Sialia sialis*) during the embryonic period, compared to high doses or control chicks [36]. After an injection of 50 ng of testosterone per yolk, Japanese quail chicks have been shown to be less fearful than controls [37], whereas, after an injection of 25 ng, chicks were more emotive [38]. Second, the method used for testosterone supplementation (injection vs maternal deposition) may be involved in these conflicting effects. Originally, steroids are not uniformly distributed in the yolk of freshly laid eggs, and vary across yolk layers, with high oestradiol concentrations in the centre, high androstenedione and testosterone concentrations in the middle and high progesterone concentrations in the peripheral layer of the yolk sphere [55], [56], [58]. As yolk layers seem to persist at least during the beginning of the incubation period, embryos may thus be exposed to temporal variations of the availability of these hormones during embryonic development [58]. In a recent study, von Engelhardt *et al*. [39] showed that a bolus injection of steroid hormones (dissolved in sesame oil) into the yolk concentrated near the area where the embryo develops and that, after 6 days of incubation, hormones were still not evenly distributed throughout the yolk. Thus, following an injection, the embryo, on the one hand, may not be exposed to the hormone at a proper time during its development and, on the other hand, may be exposed to a super-physiological dose of the hormone. Observed effects on offspring following a yolk hormone injection may therefore not reflect the observed effects on offspring following natural exposition to hormones of maternal origin.

To understand the variability of all these results, we must first grasp how maternal steroid effects on the offspring are mediated. However, we currently do not know how yolk steroids are assimilated, how and where they are metabolised, and when, where and how they act [40]. Yolk androgens affect various important physiological axes like, for example, the hypothalamus-pituitary-gonadal axis or the hypothalamus-pituitary-adrenal axis [40]. Modulation of these axes could explain behavioural differences that can be observed in offspring in the short and/or long term.

A chick's development appears to be strongly influenced by its prenatal exposition to maternal steroid hormones. Intra- and inter-individual variations of maternal hormonal levels in eggs observed in some bird species could thus reflect an adaptive significance of hormone deposition in eggs. In altricial and semi-precocial species, enhanced exposure to maternal androgens decreases incubation duration [33], [91] and stimulates begging behaviour [89], [91] and the growth of the hatching muscle (musculus complexus) involved in begging [90]. Both these effects may explain the finding that chicks then obtain a greater share of the food [91] and grow faster [33], [34], [89]. In these species, increase of egg androgen levels over the laying sequence of a clutch can thus be interpreted as a means to reduce the effect of hatching asynchrony on survival of the last chicks to hatch [18], [23]. Similarly, the increase of egg androgen levels in females subject to a high social density could prepare chicks for a high level of competition in this environment [19]. Indeed, yolk androgen may have long-term effects on a bird's behaviour, increasing overall activity and the frequency of aggressive and sexual displays [18], [33], [89], [91]–[93], influencing social dominance among nest-mates [18], and modulating neophobic responses [94]. As personality and behaviour can be important factors in determining survival and reproductive success [95], [96], egg androgens could have important fitness consequences. The adaptive consequences of maternally-derived egg androgens seem to be more difficult to evaluate in precocial species. Indeed, the hatching of precocial chicks is synchronised [97] and they are less submitted to intra-clutch competition for food, as they forage by themselves. Daisley *et al.* [37] found that a testosterone injection into quail eggs increased chicks' growth and shifted individual behavioural phenotype towards “bold” or “proactive”. These authors hypothesised that these characteristics could favour early clutch dispersion, but could also be associated with relatively poor survival rates, proactive profiles inducing potentially higher predation risks [37]. Our results showed that the eggs of females kept in unstable groups showed higher levels of testosterone and that their chicks expressed a higher level of fearfulness. Higher levels of fearfulness in chicks could help their mothers to maintain strong clutch cohesion and to restore clutch cohesion after, for example, agonistic interactions with unknown conspecifics. A previous study showed that experimental enhancement of testosterone levels in northern bobwhite quail's (*Colinus virginianus*) eggs increased fearfulness in chicks and also affected chicks' postnatal auditory preference. Indeed, the postnatal preference for familiar maternal calls (heard 24 h before hatching) of chicks from enriched eggs was stronger than that of chicks from non-treated eggs. Treated chicks moved more quickly towards the speaker broadcasting familiar maternal calls and stayed longer close to it. Moreover, treated embryos required only half the exposure time usually needed to develop a significant postnatal auditory preference [98]. Thus, in an aversive environment, having chicks that are more fearful and more sensitive to maternal calls could favour clutch cohesion and, potentially, clutch survival.

Understanding the mechanisms of prenatal maternal effects in birds requires analysing the “natural” modifications of yolk hormonal components, which are modulated by females. Moreover, although testosterone is an excellent candidate for implementing prenatal effects in birds, other sexual steroids [61] and other components of maternal origin may also play an important role in these effects. Birds' egg yolks contain many components of maternal origin, such as antioxidants, immunoglobulin or thyroid hormone that seem to have an effect on offspring growth and immunity [20]. Moreover, recent studies showed the potential role of egg corticosterone (the main glucocorticoid in birds) in prenatal maternal effects. Indeed, egg corticosterone levels seem to be influenced by the laying female's environment [99]–[101], and to affect offspring development [102], [103]. Thus, a variety of mechanisms, and not just a single pathway, could be involved in the observed prenatal maternal effects. Moreover, a better understanding of prenatal maternal effects in birds is conditional on a technical challenge, which is to improve quantification of egg hormonal levels (some cross-reactions could occur) [104] and the analysis of relationships between female plasma and egg hormonal levels [40].

In conclusion, our study shows that laying females' social environment directly influences their egg steroid levels and affects their offspring's somatic and behavioural development. Thus, even when embryos develop outside their mothers' body, they can be strongly influenced during the prenatal stage just like mammalian embryos. Eggs provide bird embryos with a complex environment that can reflect their mothers' living conditions, opening a way for transgenerational effects and providing a powerful source of behavioural variability in natural populations.

## References

[1] Jablonka E, Lamb MJ (2005). Evolution in Four Dimensions: Genetic, Epigenetic, Behavioral, and Symbolic Variation in the History of Life..

[2] Bertin A, Richard-Yris MA (2004). Mothers' fear of human affects the emotional reactivity of young in domestic Japanese quail.. Appl Anim Behav Sci.

[3] Francis DD, Champagne FA, Liu D, Meaney MJ (1999). Maternal care, gene expression, and the development of individual differences in stress reactivity.. Ann N Y Acad Sci.

[4] Calatayud F, Coubard S, Belzung C (2004). Emotional reactivity in mice may not be inherited but influenced by parents.. Physiol Behav.

[5] Bertin A, Richard-Yris MA (2005). Mothering during early development influences subsequent emotional and social behaviour in Japanese quail.. J Exp Zool A.

[6] Formanek L, Houdelier C, Lumineau S, Bertin A, Richard-Yris MA (2008). Maternal epigenetic transmission of social motivation in birds.. Ethology.

[7] Francis DD, Diorio J, Liu D, Meaney MJ (1999). Nongenomic transmission across generations of maternal behavior and stress responses in the rat.. Science.

[8] Kendrick KM, Haupt MA, Hinton MR, Broad KD, Skinner JD (2001). Sex Differences in the Influence of Mothers on the Sociosexual Preferences of Their Offspring.. Horm Behav.

[9] Champagne F (2008). Epigenetic mechanisms and the transgenerational effects of maternal care.. Front Neuroendocrinol.

[10] Liu D, Diorio J, Day J, Francis D, Meaney M (2000). Maternal care, hippocampal synaptogenesis and cognitive development in rats.. Nat Neurosci.

[11] Formanek L, Richard-Yris MA, Houdelier C, Lumineau S (2009). Epigenetic maternal effects on endogenous rhythms in precocial birds.. Chronobiol Int.

[12] Braastad B (1998). Effects of prenatal stress on behaviour of offspring of laboratory and farmed mammals.. Appl Anim Behav Sci.

[13] Welberg LA, Seckl JR (2001). Prenatal stress, glucocorticoids and the programming of the brain.. J Neuroendocrinol.

[14] Kaiser S, Sachser N (2005). The effects of prenatal social stress on behaviour: mechanisms and function.. Neurosci Biobehav Rev.

[15] Dloniak SM, French JA, Holekamp KE (2006). Rank-related maternal effects of androgens on behaviour in wild spotted hyaenas.. Nature.

[16] Barbazanges A, Piazza PV, Le Moal M, Maccari S (1996). Maternal glucocorticoid secretion mediates long-term effects of prenatal stress.. J Neurosci.

[17] Wewers D, Kaiser S, Sachser N (2005). Application of an antiandrogen during pregnancy infantilizes the male offsprings' behaviour.. Behav Brain Res.

[18] Schwabl H (1993). Yolk is a source of maternal testosterone for developing birds.. Proc Natl Acad Sci U S A.

[19] Groothuis TGG, Müller W, von Engelhardt N, Carere C, Eising CM (2005). Maternal hormones as a tool to adjust offspring phenotype in avian species.. Neurosci Biobehav Rev.

[20] Gil D (2008). Hormones in Avian Eggs: Physiology, Ecology and Behavior.. Adv Stud Behav.

[21] Schwabl H (1997). The contents of maternal testosterone in house sparrow Passer domesticus eggs vary with breeding conditions.. Naturwissenschaften.

[22] Reed W, Vleck C (2001). Functional significance of variation in egg-yolk androgens in the American coot.. Oecologia.

[23] Groothuis TGG, Schwabl H (2002). Determinants of within- and among-clutch variation in levels of maternal hormones in Black-Headed Gull eggs.. Funct Ecol.

[24] Mazuc J, Bonneaud C, Chastel O, Sorci G (2003). Social environment affects female and egg testosterone levels in the house sparrow (Passer domesticus).. Ecol Lett.

[25] Whittingham LA, Schwabl H (2002). Maternal testosterone in tree swallow eggs varies with female aggression.. Anim Behav.

[26] Müller W, Eising CM, Dijkstra C, Groothuis TGG (2002). Sex differences in yolk hormones depend on maternal social status in Leghorn chickens (Gallus gallus domesticus).. Proc R Soc B.

[27] Tanvez A, Parisot M, Chastel O, Leboucher G (2008). Does maternal social hierarchy affect yolk testosterone deposition in domesticated canaries?. Anim Behav.

[28] Gil D, Graves J, Hazon N, Wells A (1999). Male Attractiveness and Differential Testosterone Investment in Zebra Finch Eggs.. Science.

[29] Gil D, Leboucher G, Lacroix A, Cue R, Kreutzer M (2004). Female canaries produce eggs with greater amounts of testosterone when exposed to preferred male song.. Horm Behav.

[30] Kingma SA, Komdeur J, Vedder O, von Engelhardt N, Korsten P (2009). Manipulation of male attractiveness induces rapid changes in avian maternal yolk androgen deposition.. Behav Ecol.

[31] Sockman KW, Schwabl H (2000). Yolk androgens reduce offspring survival.. Proc R Soc B.

[32] Eising CM, Müller W, Dijkstra C, Groothuis TGG (2003). Maternal androgens in egg yolks: relation with sex, incubation time and embryonic growth.. Gen Comp Endocrinol.

[33] Eising CM, Eikenaar C, Schwabl H, Groothuis TGG (2001). Maternal androgens in black-headed gull (Larus ridibundus) eggs: consequences for chick development.. Proc R Soc B.

[34] Pilz KM, Quiroga M, Schwabl H, Adkins-Regan E (2004). European starling chicks benefit from high yolk testosterone levels during a drought year.. Horm Behav.

[35] Groothuis TGG, Eising CM, Dijkstra C, Müller W (2005). Balancing between costs and benefits of maternal hormone deposition in avian eggs.. Biol Lett.

[36] Navara KJ, Hill GE, Mendonca MT (2005). Variable effects of yolk androgens on growth, survival, and immunity in eastern bluebird nestlings.. Physiol Biochem Zool.

[37] Daisley J, Bromundt V, Möstl E, Kotrschal K (2005). Enhanced yolk testosterone influences behavioral phenotype independent of sex in Japanese quail chicks Coturnix japonica.. Horm Behav.

[38] Okuliarová M, Škrobánek P, Zeman M (2007). Effect of Increasing Yolk Testosterone Levels on Early Behaviour in Japanese Quail Hatchlings.. Acta Vet Brno.

[39] von Engelhardt N, Henriksen R, Groothuis TGG (2009). Steroids in chicken egg yolk: Metabolism and uptake during early embryonic development.. Gen Comp Endocrinol.

[40] Groothuis TGG, Schwabl H (2008). Hormone-mediated maternal effects in birds: mechanisms matter but what do we know of them?. Philos Trans R Soc B.

[41] Schweitzer C, Poindron P, Arnould C (2009). Social motivation affects the display of individual discrimination in young and adult Japanese quail (Coturnix japonica).. Dev Psychobiol.

[42] Schweitzer C, Houdelier C, Lumineau S, Lévy F, Arnould C (2010). Social motivation does not go hand in hand with social bonding between two familiar Japanese quail chicks, Coturnix japonica.. Anim Behav.

[43] Edens FW, Bursian SJ, Holladay SD (1983). Grouping in Japanese quail. 1. Agonistic behavior during feeding.. Poult Sci.

[44] Edens FW (1987). Manifestations of social stress in grouped Japanese quail.. Comp Biochem Physiol A.

[45] Edens FW (1987). Agonistic behavior and neurochemistry in grouped Japanese quail.. Comp Biochem Physiol A.

[46] Mills AD, Jones RB, Faure JM, Williams JB (1993). Responses to isolation in Japanese quail genetically selected for high or low sociality.. Physiol Behav.

[47] Jones RB (1996). Fear and Adaptability in Poultry: Insights, Implications and Imperatives.. World Poul Sci.

[48] Hirschenhauser K, Wittek M, Johnston P, Möstl E (2008). Social context rather than behavioral output or winning modulates post-conflict testosterone responses in Japanese quail (Coturnix japonica).. Physiol Behav.

[49] Altmann J (1974). Observational Study of Behavior: Sampling Methods.. Behaviour.

[50] Martin P, Bateson P (1993). Measuring behaviour, an introduction guide..

[51] Romero LM, Reed JM (2005). Collecting baseline corticosterone samples in the field: is under 3 min good enough?. Comp Biochem Physiol A.

[52] Hazard D, Faure JM, Guéméné D (2004). Effects of sampling sites, photoperiod, and age on adrenal responsiveness in Japanese quail..

[53] Etches RJ (1976). A radioimmunoassay for corticosterone and its application to the measurement of stress in poultry.. Steroids.

[54] Sauveur B, Picard M, Wells RG, Belyavin CG (1987). Environmental effects on egg quality.. Egg quality: current problems and recent advances.

[55] Möstl E, Spendier H, Kotrschal K (2001). Concentration of immunoreactive progesterone and androgens in the yolk of hens' eggs (Gallus domesticus).. Wien Tierarztl Mschr.

[56] Hackl R, Bromundt V, Daisley J, Kotrschal K, Möstl E (2003). Distribution and origin of steroid hormones in the yolk of Japanese quail eggs (Coturnix coturnix japonica).. J Comp Physiol [B].

[57] Bertin A, Richard-Yris MA, Houdelier C, Lumineau S, Möstl E (2008). Habituation to humans affects yolk steroid levels and offspring phenotype in quail.. Horm Behav.

[58] Lipar JL, Ketterson ED, Nolan V, Casto JM (1999). Egg yolk layers vary in the concentration of steroid hormones in two avian species.. Gen Comp Endocrinol.

[59] Palme R, Möstl E (1993). Biotin-streptavidin enzyme immunoassay for the determination of oestrogens and androgens in boar faeces..

[60] Hirschenhauser K, Möstl E, Kotrschal K (1999). Seasonal Patterns of Sex Steroids Determined from Feces in Different Social Categories of Greylag Geese (Anser anser).. Gen Comp Endocrinol.

[61] Hegyi G, Schwabl H (2010). Do different yolk androgens exert similar effects on the morphology or behaviour of Japanese quail hatchlings Coturnix japonica?. J Avian Biol.

[62] Forkman B, Boissy A, Meunier-Salaün MC, Canali E, Jones RB (2007). A critical review of fear tests used on cattle, pigs, sheep, poultry and horses.. Physiol Behav.

[63] Jones RB, Mills AD, Faure JM (1991). Genetic and Experiential Manipulation of Fear-Related Behavior in Japanese Quail Chicks (Coturnix coturnix japonica).. J Comp Psychol.

[64] Archer J, Bateson P, Klopfer P (1976). The organization of aggression and fear in vertebrates..

[65] Mills AD, Faure JM (1986). The estimation of fear in domestic quail: correlations between various methods and measures.. Biol Behav.

[66] Jones RB, Zayan R, Ducan IJH (1987). The assessment of fear in the domestic fowl..

[67] Launay F (1993). Conséquences comportementales et physiologiques de sélections pour l'émotivité et l'attraction sociale chez la caille japonaise (Coturnix c. japonica)..

[68] Jones RB, Merry BJ (1988). Individual or paired exposure of domestic chicks to an open field: Some behavioural and adrenocortical consequences.. Behav Proc.

[69] Deminière JM, Piazza PV, Guegan G, Abrous N, Maccari S (1992). Increased locomotor response to novelty and propensity to intravenous amphetamine self-administration in adult offspring of stressed mothers.. Brain Res.

[70] Vallee M, Mayo W, Dellu F, Le Moal M, Simon H (1997). Prenatal Stress Induces High Anxiety and Postnatal Handling Induces Low Anxiety in Adult Offspring: Correlation with Stress-Induced Corticosterone Secretion.. J Neurosci.

[71] Formanek L, Houdelier C, Lumineau S, Bertin A, Cabanès G (2008). Selection of social traits in juvenile Japanese quail affects adults' behaviour.. Appl Anim Behav Sci.

[72] Summers CH, Watt MJ, Ling TL, Forster GL, Carpenter RE (2005). Glucocorticoid interaction with aggression in non-mammalian vertebrates: Reciprocal action.. Eur J Pharmacol.

[73] Mills AD, Crawford LL, Domjan M, Faure JM (1997). The behavior of the Japanese or domestic quail Coturnix japonica.. Neurosci Biobehav Rev.

[74] Guyomarc'h JC, Combreau O, Puigcerver M, Fontoura P, Aebischer N (1998). Coturnix coturnix Quail. In: BWP Update vol 2..

[75] Aubrais O, Hemon Y, Guyomarc'h JC (1986). Habitat and use of space in the common quail (Coturnix coturnix corturnix) at the onset of the breeding period.. Gibier Faune Sauvage.

[76] Guyomarc'h JC, Hemon Y, Aubrais O, Saint-Jalme M (1986). Approche éthologique de la structure et du fonctionnement des populations reproductrices de cailles des blés (1).. BM ONC.

[77] Nichols CR (1991). A comparison of the reproductive and behavioural differences in feral and domestic Japanese quail..

[78] Hemon Y, Saint-Jalme M, Guyomarc'h JC (1988). Structure et fonctionnement des populations reproductrices “françaises” de cailles des blés.. BM ONC.

[79] Pilz KM, Smith HG (2004). Egg yolk androgen levels increase with breeding density in the European Starling, Sturnus vulgaris.. Funct Ecol.

[80] Wingfield J, Short RV, Balaban E (1994). Hormone-behavior interactions and mating systems in male and female birds.. The differences between the sexes.

[81] Schwabl H (1996). Environment modifies the testosterone levels of a female bird and its eggs.. J Exp Zool.

[82] Badyaev AV, Schwabl H, Young RL, Duckworth RA, Navara KJ (2005). Adaptive sex differences in growth of pre-ovulation oocytes in a passerine bird.. Proc R Soc B.

[83] Navara KJ, Siefferman LM, Hill GE, Mendonça MT (2006). Yolk androgens vary inversely to maternal androgens in Eastern Bluebirds: an experimental study.. Funct Ecol.

[84] Pilz KM, Smith HG, Sandell MI, Schwabl H (2003). Interfemale variation in egg yolk androgen allocation in the European starling: do high-quality females invest more?. Anim Behav.

[85] Williams TD, Kitaysky AS, Vézina F (2004). Individual variation in plasma estradiol-17[beta] and androgen levels during egg formation in the European starling Sturnus vulgaris: implications for regulation of yolk steroids.. Gen Comp Endocrinol.

[86] Marshall RC, Leisler B, Catchpole CK, Schwabl H (2005). Male song quality affects circulating but not yolk steroid concentrations in female canaries (Serinus canaria).. J Exp Biol.

[87] Rubolini D, Romano M, Martinelli R, Saino N (2006). Effects of elevated yolk testosterone levels on survival, growth and immunity of male and female yellow-legged gull chicks.. Behav Ecol Sociobiol.

[88] von Engelhardt N, Carere C, Dijkstra C, Groothuis TGG (2006). Sex-specific effects of yolk testosterone on survival, begging and growth of zebra finches.. Proc R Soc B.

[89] Schwabl H (1996). Maternal testosterone in the avian egg enhances postnatal growth.. Comp Biochem Physiol A.

[90] Lipar JL, Ketterson ED (2000). Maternally derived yolk testosterone enhances the development of the hatching muscle in the red-winged blackbird Agelaius phoeniceus.. Proc R Soc B.

[91] Eising CM, Groothuis TGG (2003). Yolk androgens and begging behaviour in black-headed gull chicks: an experimental field study.. Anim Behav.

[92] Eising CM, Müller W, Groothuis TGG (2006). Avian mothers create different phenotypes by hormone deposition in their eggs.. Biol Lett.

[93] Rubolini D, Romano M, Martinelli R, Leoni B, Saino N (2006). Effects of prenatal yolk androgens on armaments and ornaments of the ring-necked pheasant.. Behav Ecol Sociobiol.

[94] Tobler M, Sandell MI (2007). Yolk testosterone modulates persistence of neophobic responses in adult zebra finches, Taeniopygia guttata.. Horm Behav.

[95] Wilson DS, Clark AB, Coleman K, Dearstyne T (1994). Shyness and boldness in humans and other animals.. Trends Ecol Evol.

[96] Dingemanse NJ, Réale D (2005). Natural selection and animal personality.. Behaviour.

[97] Vince MA (1966). Artificial acceleration of hatching in quail embryos.. Anim Behav.

[98] Bertin A, Richard-Yris MA, Möstl E, Lickliter R (2009). Increased yolk testosterone facilitates prenatal perceptual learning in Northern bobwhite quail (Colinus virginianus).. Horm Behav.

[99] Hayward LS, Satterlee DG, Wingfield JC (2005). Japanese quail selected for high plasma corticosterone response deposit high levels of corticosterone in their eggs.. Physiol Biochem Zool.

[100] Saino N, Romano M, Ferrari RP, Martinelli R, Møller AP (2005). Stressed mothers lay eggs with high corticosterone levels which produce low-quality offspring.. J Exp Zool A.

[101] Downing JA, Bryden WL (2008). Determination of corticosterone concentrations in egg albumen: a non-invasive indicator of stress in laying hens.. Physiol Behav.

[102] Hayward LS, Wingfield JC (2004). Maternal corticosterone is transferred to avian yolk and may alter offspring growth and adult phenotype.. Gen Comp Endocrinol.

[103] Janczak AM, Braastad B, Bakken M (2006). Behavioural effects of embryonic exposure to corticosterone in chickens.. Appl Anim Behav Sci.

[104] Rettenbacher S, Möstl E, Groothuis TGG (2009). Gestagens and glucocorticoids in chicken eggs.. Gen Comp Endocrinol.

